# Rhythm Disturbances in Post-Acute COVID-19 Syndrome in Young Men without Pre-Existing Known Cardiovascular Disease—A Case Series

**DOI:** 10.3390/biomedicines11041146

**Published:** 2023-04-11

**Authors:** Ciprian Ilie Rosca, Horia Silviu Branea, Abhinav Sharma, Violeta Ariana Nicoras, Claudia Borza, Daniel Florin Lighezan, Stelian I. Morariu, Nilima Rajpal Kundnani

**Affiliations:** 1 Center of Advanced Research in Cardiovascular Pathology and Haemostasis, Department of Internal Medicine I—Medical Semiotics I, “Victor Babes” University of Medicine and Pharmacy, 300041 Timisoara, Romania; 2 Department of Internal Medicine I—Medical Semiotics II, “Victor Babes” University of Medicine and Pharmacy, 300041 Timisoara, Romania; 3 Department of Cardiology—Internal Medicine and Ambulatory Care, Prevention and Cardiovascular Recovery, “Victor Babes” University of Medicine and Pharmacy, 300041 Timisoara, Romania; 4 General Medicine Faculty, “Vasile Goldis” West University, 473223 Arad, Romania; 5 Center for Translational Research and Systems Medicine, Department of Pathophysiology, “Victor Babes” University of Medicine and Pharmacy, 300041 Timisoara, Romania

**Keywords:** COVID-19, post-acute COVID-19 syndrome, cardiovascular changes in post-acute COVID-19 syndrome, non-sustained ventricular tachycardia, paroxysmal atrial fibrillation

## Abstract

Current data indicate the existence of post-acute COVID-19 syndrome frequently expressing as cardiovascular and respiratory health issues. The long-term evolution of these complications is not yet fully known or predictable. Among the most common clinical manifestations of post-acute COVID-19 syndrome are dyspnea, palpitations, and fatigue, in most cases being transient and without underlying any morphological or functional changes. A single-center retrospective observational study was performed on cases that had presented with new-onset cardiac symptoms post-COVID-19 infection. Records of three male patients without pre-existing chronic cardiovascular pathology who had presented for dyspnea, fatigue, and palpitations around four weeks post-COVID-19 acute phase were studied in detail. The three post-COVID-19 cases exhibited arrhythmic complications after completely healing from the acute phase of the infection. Palpitations, along with chest pain, and possible aggravation or appearance of dyspnea, with syncopal episodes, were found to be present. All the three cases were non-vaccinated against COVID-19 infection. Isolated case reports showing arrhythmic complications such as atrial fibrillation and ventricular tachycardia on a small number of patients with these complications indicate the need for arrhythmic evaluation of large groups of patients in the post-acute stage of the COVID-19 syndrome for a better understanding of the phenomenon and implicitly better care of these patients. It would also be useful to evaluate large groups of patients divided into vaccinated/non-vaccinated against COVID-19 categories to determine whether vaccination per se can provide protection in the occurrence of these types of complications.

## 1. Introduction

Ever since 2019, when SARS-CoV-2 (Severe Acute Respiratory Syndrome Coronavirus) was first diagnosed in China, very little has been known about the course of the disease and its late complications [1]. Vague and uncertain medical data are slowly starting to gather, but the short-, medium- and long-term evolution of patients infected with this new virus remains unpredictable [2]. The passage of time makes it necessary to use a common language in terms of communications related to this new disease, so recommendations are made regarding the ICD codes used for this purpose [3,4]. The persistence of symptoms far from the initial infection with COVID-19 (at least four weeks after the onset of the infection) have been reported even for previously asymptomatic or mildly symptomatic infections [2,5]. Respiratory involvement is the most common, but the kidneys, heart or nervous system can also be affected by this infection, culminating in a multi-organ involvement [6]. Regarding cardiac damage in COVID-19 patients, venous thromboembolism, myocardial ischemia and myocardial infarction, isolated cases of myocarditis (some cases with a fatal outcome), and acute heart failure with cardiogenic shock have been reported during the acute phase of the infection, in addition to reports of sustained ventricular tachycardia and atrial arrhythmias [7,8,9,10,11]. In post-acute COVID-19 syndrome (PACS), symptoms such as chest pain, palpitations, and fatigue can persist or appear after a prolonged period of time [12,13] and may be accompanied later on by supraventricular or ventricular arrhythmias, cardiomyopathies, congestive heart failure, arterial hypertension or pericarditis as well as cardiomegaly or bradycardia although these are less commonly witnessed [14,15,16]. Cardiovascular manifestations of PACS are frequently seen in patients with pre-existing cardiovascular pathology [16,17]. Furthermore, the structural and functional changes appearing in PACS seem to be persistent over time as documented by some studies [18,19]. The three cases presented draw attention to the possibility of the existence of arrhythmic complications with malignant potential in patients in the post-acute phase of COVID-19 syndrome in patients with unknown cardiovascular pathology.

## 2. Materials and Methods

We present the cases of three young men, without previous cardiovascular pathology, who presented for evaluation in the cardiology outpatient clinic for a recent onset of cardiac symptoms. They were selected from the patients who were evaluated in our medical unit between July and December 2021 for cardiovascular symptoms onset post-COVID-19. These three patients met the following criteria: post-acute COVID-19 infection previously healthy men without known cardiovascular pathology and with symptoms related to cardiovascular disease that were not present before COVID-19 infection and which presented on Holter ECG monitoring, and arrhythmias other than supraventricular ones. None of them were vaccinated against COVID-19 (but that finding was not a selection criterion). Among these patients, there was no family history of cardio-vascular pathology such as dilated cardiomyopathy, chronic coronary syndrome, or arrhythmias. Patients underwent (as a routine cardiological evaluation of a patient who is presented for dyspnea, fatigue, chest pain, and palpitations) general clinical examination, resting ECG, Holter EKG/24 h monitoring, 2D, M-mode and Doppler echocardiography, laboratory data, and one case underwent cardiac MRI (the other two patients did not undergo cardiac MRI due to financial constraints, as it is not covered by the National Health Insurance system). None of these patients were hospitalized during the acute phase of COVID-19 infection or for PACS and were treated in the outpatient department (OPD). Data were collected and analyzed.

## 3. Presentation of Cases

### 3.1. Case 1

A 39-year-old previously healthy male patient, without any family history of cardiovascular diseases such as arrhythmia or dilatative cardiomyopathies, etc., was found to be positive for COVID-19 based on an RT-PCR SARS-COV2 test on 30 May 2021. During the entire phase of active infection, he presented mild common symptoms such as rhinorrhea, myalgia, fatigue, and a fever of 37.9 °C. which were remitted from the third day of the evolution of the acute infection. Five weeks after the acute phase of the COVID-19 infection (July 2021), he was referred to the cardiology ambulatory by his family doctor due to dyspnea, various episodes of irregular paroxysmal palpitations, severe fatigue even at rest, and complaints of intermittent unspecific chest pain, symptoms that were never experienced until the COVID-19 infection. The general condition was found to be stable; further details are presented in Table 1:

The resting electrocardiogram (ECG) revealed paroxysmal atrial fibrillation, PVCs with a monomorphic appearance of right bundle branch block (RBBB), isolated, bigeminy, triplet, and non-sustained ventricular tachycardias (nsVTs).

**Figure 1:**
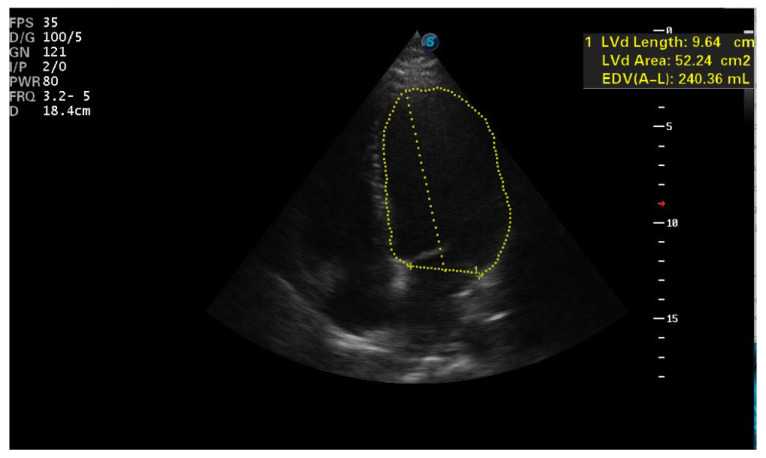
Case 1—TTE left ventricle tele-diastolic volume evaluation (yellow dotted line).

**Figure 2:**
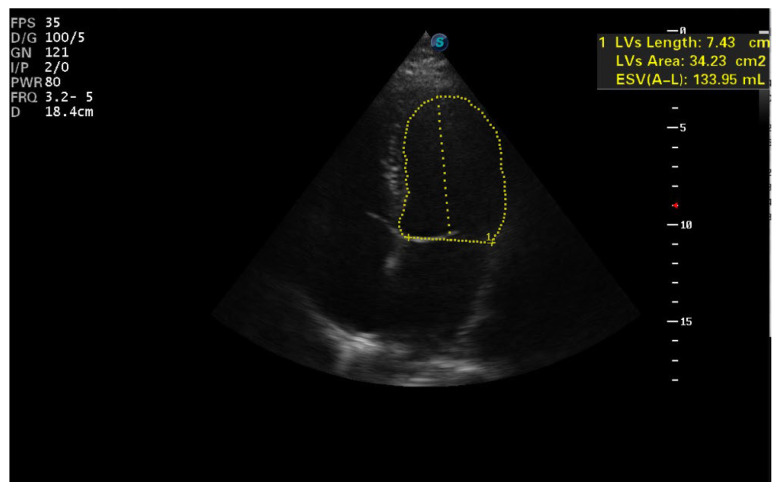
Case 1—TTE left ventricle tele-systolic volume evaluation (yellow dotted line).

**Figure 3:**
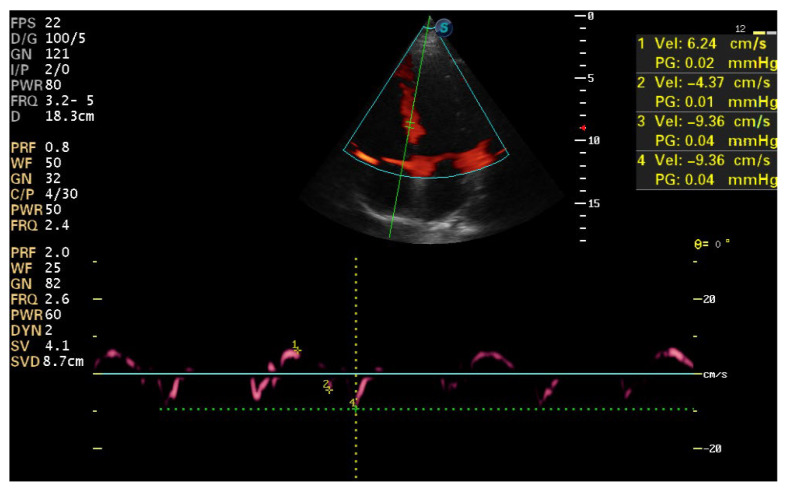
Case 1—Tissue Doppler Imaging septum evaluation (1-S wave, 2-e’ wave, 3,4-a’ wave).

Transthoracic echocardiography (TTE) revealed a left ventricle (LV) with systolic dysfunction (left ventricle ejection fraction (LVEF) 43%) and normal diastolic function, significant interventricular septum hypertrophy (IVS) and mild posterior wall hypertrophy (LVPW), dilated cavity. Bi-atrial dilatation. There was mild dilatation of the right ventricle. Moderate mitral regurgitation due to dilatation of the mitral annulus, and moderate functional pulmonary insufficiency (Figure 1, Figure 2 and Figure 3). Table 2 shows the main echocardiographic parameters.

**Figure 4:**
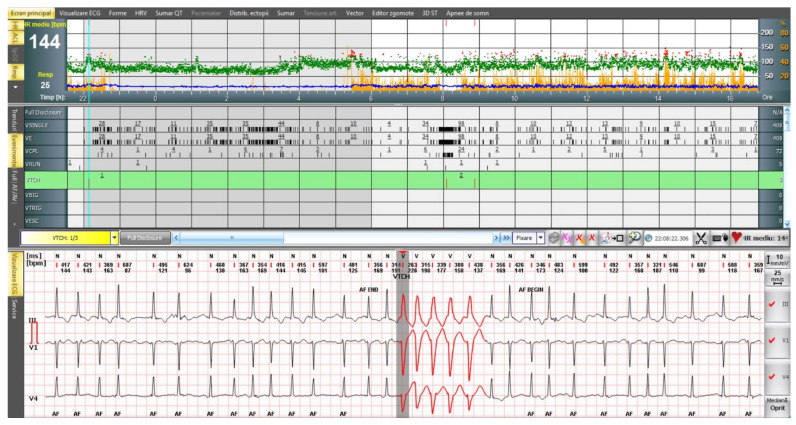
Case 1—ECG monitoring showing atrial fibrillation and non-sustained ventricular tachycardia (AF—atrial fibrillation, VTCH—ventricular tachycardia). QRS axis changes, with wide QRS complex.

**Figure 5:**
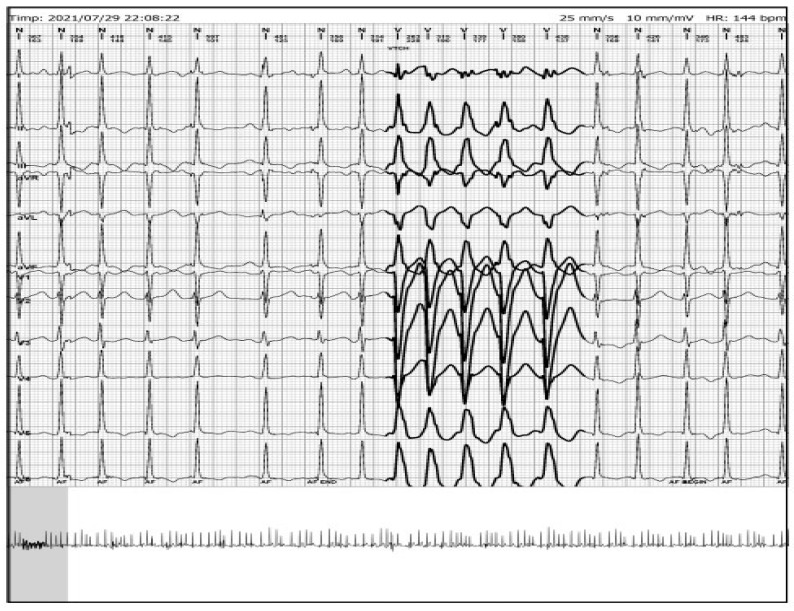
Case 1— Additional ECG monitoring showing atrial fibrillation and non-sustained ventricular tachycardia. QRS axis changes, with wide QRS complex.

Holter ECG (12 ch-recorder) monitoring revealed: The average heart rate was 88 bpm. The minimum heart rate was 53 bpm. The maximum heart rate was 152 bpm. Ventricular results: There were a total of 581 (0.58%) ectopic beats. These comprised 408 (70.22%) single beats, 72 (24.78%) couplets, 5 (2.58%) runs, 3 (2.41%) tachycardiac events. Fastest tachycardia episode: 4 beats, 211 bpm. Longest tachycardia episode: 5 beats, 185 bpm. Supraventricular results: There were a total of 13,820 (13.78%) beats. These comprised 6477 (46.87%) single beats, 2000 (28.94%) couplets, 477 (10.35%) runs, 312 (13.84%) tachycardiac events, and there were 22 (0.5%) bigeminy events. Fastest tachycardia episode: 5 beats, 218 bpm. Longest tachycardia episode: 25 beats, 145 bpm. Rhythm results: atrial fibrillation was detected in 12.32% (2 h 22 min) of the total monitoring time (Figure 4 and Figure 5).

Considering the TTE aspects identified as well as the rhythm disorders detected by the ECG, in conjunction with the epidemiological context, it was recommended to perform a cardiac MRI.

Treatment was initiated with amiodarone 3 × 600 mg/day for 10 days then 1 pc/day, bisoprolol 5 mg/day, ramipril 5 mg/day, empagliflozin 10 mg/day, apixaban 2 × 5 mg/day, and alprazolam 2 × 0.25 mg/day.

Three months after the first presentation for cardiac symptoms, the patient’s general condition was good, without describing arrhythmic episodes, fatigue, or dyspnea, but the patient had high blood pressure values despite the use of anti-hypertensive medications; in addition, the patient showed an increased level of anxiety. Further, a follow-up Holter ECG monitoring revealed iatrogenic sinus bradycardia without rhythm or conduction disturbances. Echocardiographic evaluation indicated a decrease in the volumes of the cardiac cavities as well as the thickness of the LV walls with an overall slight improvement in LVEF.

The therapeutic scheme was readjusted by suppressing the treatment with amiodarone and increasing the dose of ramipril to 2 × 5 mg/day, keeping the rest of the medication according to the previous recommendations.

It was recommended to perform a cardiac MRI, which the patient performed at the beginning of December 2021, and which revealed cardiac MRI appearance within normal limits.

Six months after the first presentation, the patient returned for a follow-up without arrhythmic episodes or other symptoms but with high blood pressure values partially controlled by medication. Resting ECG revealed sinus rhythm with an HR of 50 bpm. Holter ECG monitoring showed persistent sinus bradycardia with a minimum HR of 35 bpm throughout the day without any arrhythmic episodes. The echocardiographic appearance showed no further improvement compared to the previous 3-month assessment. The dose of bisoprolol was reduced to 2.5 mg/day, and the ramipril was replaced by candesartan 8 mg/day, keeping the anti-coagulant treatment with apixaban 2 × 5 mg/day as well as empagliflozin 10 mg/day, and removing the treatment with alprazolam by tapering over the course of 14 days.

After 2 weeks had passed since the readjustment of the therapeutic scheme, due to the persistence of the sinus bradycardia (HR varying between 50 and 55 bpm during BP self-monitoring with the personal electronic blood pressure monitor), it was decided to discontinue the treatment with bisoprolol.

During the 1st week after stopping treatment with bisoprolol, the patient had paroxysmal episodes of rapid and irregular palpitations, associated chest pain, and dyspnea, which is why he reintroduced bisoprolol 2.5 mg/day on his own initiative, continuing the treatment with candesartan, empagliflozin, and apixaban.

At the evaluation 1 year after the first presentation, the patient was found to be completely asymptomatic, without any rhythm or conduction disturbances on Holter ECG monitoring.

### 3.2. Case 2

A 30-year-old man, without any known chronic pathology prior to COVID-19 and with no family history of arrhythmic or dilatative cardiovascular diseases, presented to our clinic with mucous rhinorrhea, pain in the throat of intensity 9/10, minimal fatigue, a fever of 38.2 °C for one day, and then afebrile. On RT-PCR SARS-COV2 testing, he was found to be positive. From the third day of presentation, he had no more symptoms. Seven weeks after the infection in December 2021, the patient presented to our clinic again complaining of marked fatigue at rest, dyspnea, many episodes of rapid and irregular palpitations with a paroxysmal character occurring both during the day and night, non-specific episodes of chest pain and two episodes of syncope that occurred before the presentation, severe anxiety, and fear of death. At presentation, the patient was hemodynamically stable; details regarding the patient are presented in Table 1.

Resting ECG showed the presence of sinus rhythm, regular HR 62 bpm, and axis of the QRS complex at 70 degrees, without changes.

**Figure 6:**
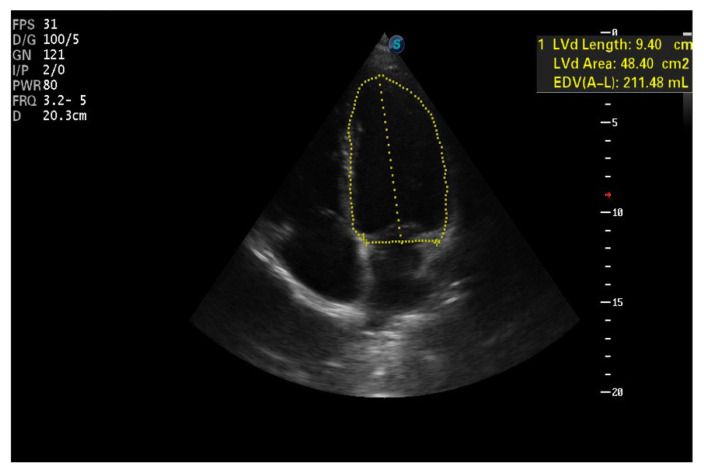
Case 2—TTE showing tele-diastolic LV volume evaluation (yellow dotted line).

**Figure 7:**
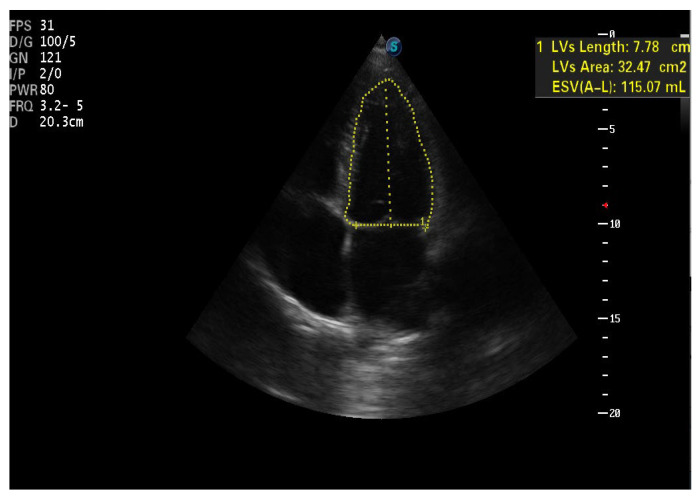
Case 2—TTE showing tele-systolic LV volume evaluation (yellow dotted line).

TTE showed LV with systolic dysfunction (LVEF = 46%) and normal diastolic function, a slightly dilated cavity, and discrete IVS hypertrophy. There was mild dilatation of the right ventricle, bi-atrial dilatation, and minimal functional tricuspid regurgitation. The main parameters of TTE are presented in Table 2 and in Figure 6 and Figure 7.

**Figure 8:**
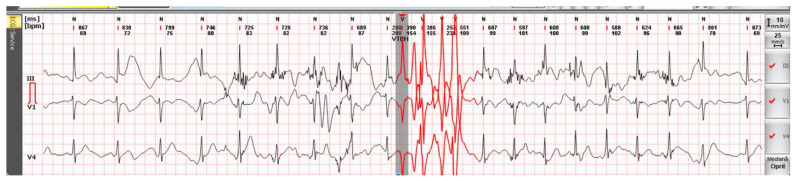
Case 2—ECG Holter monitoring capture showing ventricular tachycardia (VTCH—ventricular tachycardia).

Holter ECG monitoring (12 ch-recorder) revealed the following: The average heart rate was 80 bpm. The minimum heart rate was 43 bpm. The maximum heart rate was 149 bpm. Ventricular results: there were a total of 806 (0.7%) beats—645 (80.02%) single beats, 55 (13.65%) couplets, 6 (2.23%) runs, 7 (4.09%) tachycardiac events; there were 4 (1.49%) bigeminy events. Fastest tachycardia episode: 4 beats, 174 bpm. Longest tachycardia episode: 7 beats, 117 bpm. Supraventricular results: there were a total of 79 (0.07%) beats—18 (22.78%) single beats, 9 (22.78%) couplets, 1 (3.8%) runs, 7 (50.63%) tachycardiac events. Fastest tachycardia episode: 4 beats, 145 bpm. Longest tachycardia episode: 9 beats, 124 bpm. Rhythm results: atrial fibrillation was detected in 0.11% (0 h 01 min) of the total monitoring time (Figure 8).

Treatment was initiated with amiodarone 200 mg/day, bisoprolol 2.5 mg/day, apixaban 2 × 5 mg/day, and empagliflozin 10 mg/day.

A 3-month follow-up revealed the improvement of LV systolic function (LVEF 65%, LVTDV 108.1 ml/m^2^ bs, LVTSV 37.8 mL/m^2^bs) as well as the TDI parameters. Holter ECG monitoring was normal. However, the patient’s dyspnea persisted. Spirometry was normal. The treatment with amiodarone and empagliflozin was stopped, but the administration of bisoprolol and apixaban was continued.

### 3.3. Case 3

A previously healthy 29-year-old man with no family history of cardiac pathologies presented to our clinic after a COVID-19 infection with the following symptomatology: sore throat with maximum intensity grades as 6/10 (suddenly relieved on the 1st day after onset), moderate fatigue, and a fever of 37.9 °C for two days before becoming afebrile. He was found to be positive for COVID-19 on RT-PCR SARS-COV2 testing. After 6 weeks in January 2022, the patient presented to our unit complaining of marked fatigue at rest, dyspnea, rapid and irregular palpitations with a paroxysmal character, non-specific episodes of chest pain, and a syncopal episode. The details of the investigations are summarized in Table 1.

Resting ECG revealed sinus rhythm, irregular HR 95 bpm, QRS axis at 50°, and polyfocal PVCs with a tendency to systematize in ventricular bigeminy.

**Figure 9:**
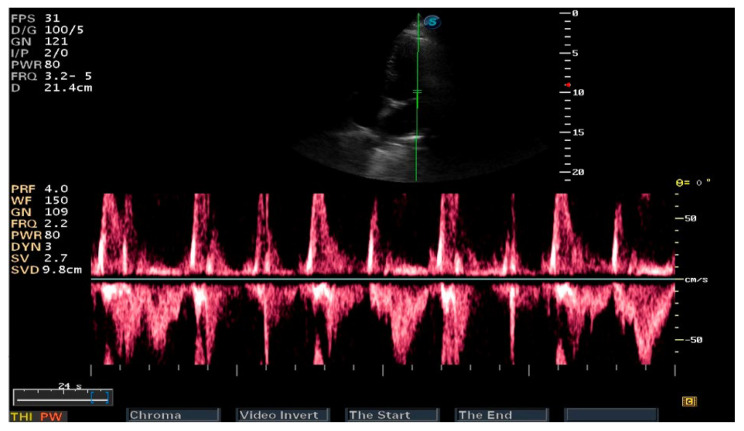
Case 3—TTE recording showing paroxysmal atrial fibrillation appeared at the time of A5C evaluation of mitral valve (A5C—apical 5 chamber).

**Figure 10:**
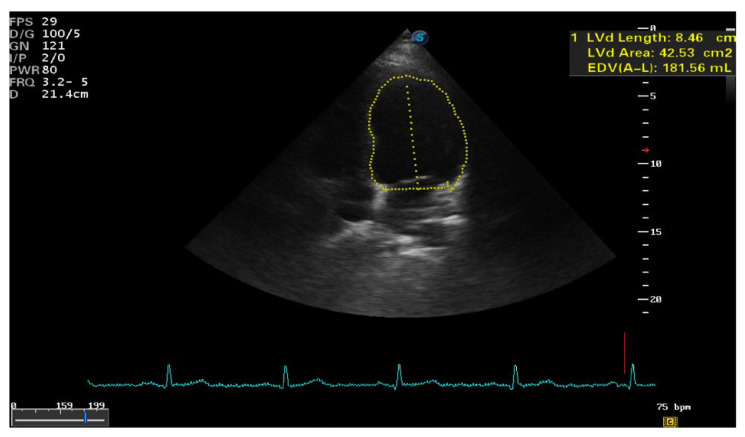
Case 3—TTE Left ventricle tele-diastolic volume evaluation (yellow dotted line).

**Figure 11:**
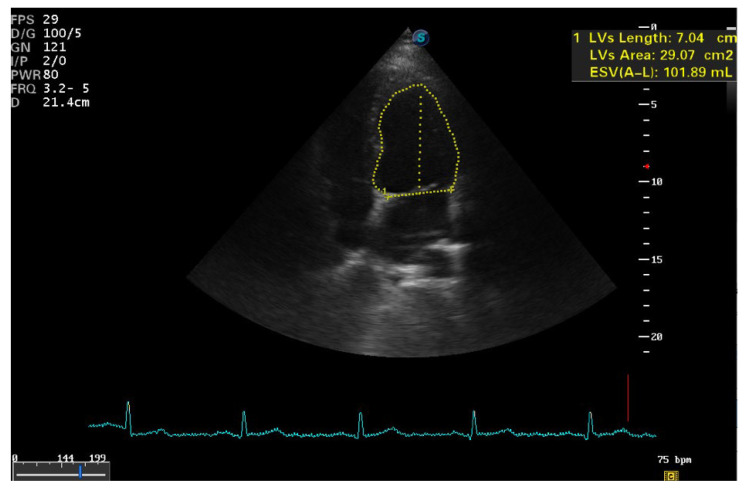
Case 3—TTE Left ventricle tele-systolic volume evaluation (yellow dotted line).

The TTE echocardiographic evaluation revealed: the LV with systolic dysfunction (LVEF = 44%), diastolic dysfunction type I, walls were in normal range, and a slightly dilated cavity as well as mild functional pulmonary regurgitation. There were no parietal kinetics disorders. The pericardial effusion was 5 mm anterior to the RV and 3 mm posterior to the LV. During the echocardiographic evaluation, there were several paroxysmal episodes of atrial fibrillation (Figure 9, Figure 10 and Figure 11).

**Figure 12:**
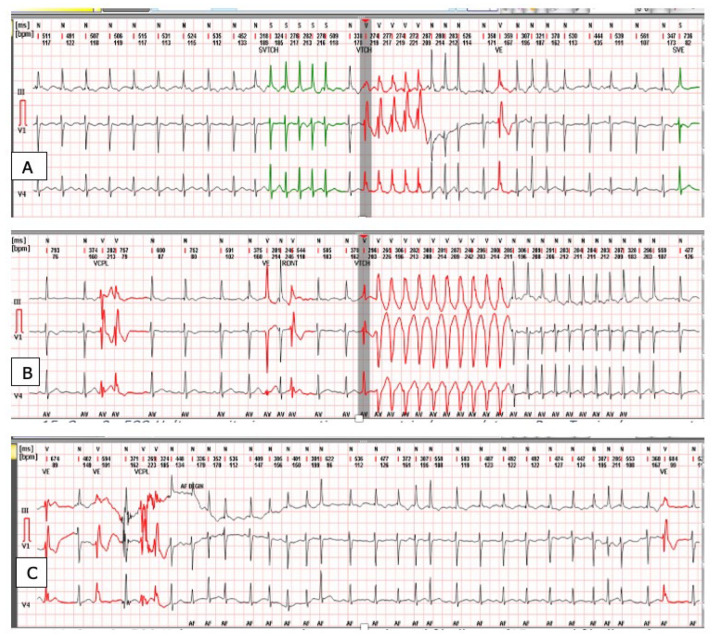
Case 3—(**A**) ECG Holter monitoring shows an episode of supraventricular tachycardia and non-sustained ventricular tachycardia (SVTCH—supraventricular tachycardia, VTC—ventricular tachycardia). (**B**) ECG Holter monitoring presenting one ventricular couplet, one R on T episode, non-sustained ventricular tachycardia, and paroxysmal atrial fibrillation (VCPL—ventricular couplet, VE—ventricular premature contraction, RONT R on T phenomenon VTCH—ventricular tachycardia). (**C**) ECG Holter monitoring with paroxysmal atrial fibrillation (AF—atrial fibrillation).

The 24 h ECG Holter monitoring revealed: The average heart rate was 101 bpm. The minimum heart rate was 64 bpm. The maximum heart rate was 196 bpm. Ventricular results: There were a total of 734 (0.49%) beats—429 (58.45%) single beats, 85 (23.16%) couplets, 25 (10.22%) runs, 11 (8.17%) tachycardiac events. Fastest tachycardia episode: 10 beats, 219 bpm. Longest tachycardia episode: 11 beats, 211 bpm. Supraventricular results: There were a total of 6636 (4.46%) beats—936 (14.1%) single beats, 379 (11.42%) couplets, 203 (9.18%) runs, and 349 (65.28%) tachycardiac events. Fastest tachycardia episode: 6 beats, 240 bpm. Longest tachycardia episode: 771 beats, 166 bpm. Rhythm results: Atrial fibrillation was detected in 0.20% (0 h 03 min) of the total monitoring time. Atrial flutter was detected in 0.46% (0 h 06 min) of the entire monitoring time (Figure 12).

Treatment was initiated with ibuprofen 1800 mg/day, colchicine 0.5 mg/day for 4 days, then 1 mg/day, amiodarone 200 mg/day, apixaban 2 × 5 mg/day, and empagliflozin 10 mg/day.

The evaluations at one week and at two weeks show a minimal echocardiographic improvement (by decreasing the amount of pericardial fluid) and by the disappearance of the patient’s symptoms. Complete resorption of pericardial fluid was achieved one month after the first presentation. After complete resorption, withdrawal from the ibuprofen treatment began by decreasing the administered dose by 300 mg/day every 3 days. The rest of the treatment remained unchanged.

On a 3-month follow-up, the patient still had complaints of persisting moderate fatigue as well as of non-specific chest pain. ECG Holter monitoring was repeated and was found to be normal. Transthoracic echocardiography showed no improvement compared to the previous evaluation. Colchicine was discontinued, while the rest of the prescription remained unchanged.

At the 6-month evaluation visit, the patient still presented with complaints of fatigue and non-specific chest pain but mild in nature, compared to the previous evaluation. ECG Holter monitoring did not identify arrhythmic or ischemic phenomena, and TTE did not show any improvement. It was decided to stop the amiodarone treatment.

Even at 1 year after the first presentation, the patient still reported fatigue and non-specific chest pain with the same echocardiographic changes as before and ECG Holter monitoring within normal limits.

## 4. Discussion

The increase in the sensitivity of the diagnostic tests used to detect the presence of the SARS-CoV-2 virus, as well as the decrease in the cost of these determinations, made it possible to identify infected cases with great accuracy [20]. Normal values of inflammation markers at the time of the first evaluation exclude the presence of an acute infectious and inflammatory process. The presence of dilation of the left cavities of the heart and the alteration of the systolic function (placing the patients in the class of heart failure with a slightly reduced ejection fraction) even in the presence of normal values of NT-pro-BNP can lead us to think of a myocarditis-type process due to acute infection with COVID-19. Likewise, the presence of arrhythmic phenomena (NSVT, AF), detected at the first evaluation but which was no longer detected at the three-month evaluation, adds to this assumption [21]. These assumptions can best explain the evolution of these patients especially against the background of the absence of diagnostic standards for myocarditis from the COVID-19 infection.

The distance of at least four weeks from the infection with COVID-19 to the moment of detection of the infection with COVID-19 suggests that these patients can be included in the category of patients with post-acute COVID-19 syndrome.

The presence of a mild form of the COVID-19 infection would make the occurrence of arrhythmic phenomena such as NSVT less likely, this type of complication being more recently associated with severe forms of this infection [22]; however, our patients were objectified to the presence of NSVT by ECG Holter monitoring showing repeated episodes of NSVT associating with them multiple PVCs that appear both isolated and bigeminy, trigeminy or in doublets.

However, cardiovascular complications in COVID-19 patients are much more frequent and of a higher grade of severity in patients with previous cardiac damage. Myocarditis and pericarditis are among the most common cardiovascular complications of the infection with COVID-19 [23,24,25] and can explain the evolution of our patients in the stage of post-acute COVID-19 syndrome.

The arrhythmic substrate represented by the cytokine storms [26,27,28] is involved in the occurrence of cardiac complications of the COVID-19 in the acute phase of the infection. New-onset atrial fibrillation during COVID-19 acute infection is associated with poor clinical outcome [29], an aspect that can also be observed in our patients, 2 of them maintaining their left ventricular ejection fraction alteration 1 year after the first evaluation.

Ventricular arrhythmias are less frequent or at least less frequently documented complications in patients in the phase of post-acute COVID-19 syndrome and could not be correlated with the subsequent evolution of these patients [16]. However, the presence of ventricular PCs is associated in some studies with an increase in the incidence and persistence of heart failure in COVID-19 positive patients [30]. In the case series, cases one and three presented a more important arrhythmic burden and systolic dysfunction of the left ventricle one year after the first assessment, unlike the second case, in which the systolic function of the left ventricle was normalized later. Similar results are documented in various studies on COVID-19 positive cases, that had systolic dysfunction of the ventricle noted [31,32,33].

Many studies document the post-COVID-19 syndrome, but few focus on young patients having no previous cardiovascular pathologies [34]. It is of utmost importance to evaluate all the cases presenting with arrhythmic changes, even if they were previously healthy individuals [35,36].

The limitation of this case series report is the lack of confirmatory MRI to rule out myocarditis (only one patient was able to perform it and had no myocarditis). Endomyocardial biopsy could be an alternative although not frequently advisable, being an invasive procedure, but the patients refused as it requires hospitalization. Myocarditis being the culprit behind the changes found in these patients is based on the clinical and paraclinical elements highlighted in our patients. The lack of paraclinical investigations at the time of diagnosis of the infection with COVID-19 did not allow us to correctly assess the mechanisms underlying the occurrence of NSVT episodes in our patients.

## 5. Conclusions

Acute and chronic long-term COVID-19 complications require large cohort studies to better understand the disease evolution. Asymptomatic or mildly symptomatic cases can present with late complications involving arrythmias and systolic dysfunction hindering the quality of life of patients. In our case series, all the three cases included had mild symptoms and fast recovery after acute COVID-19 infection but later presented with cardiac pathologies. The findings of our case series and other similar case presentations should help set protocols for follow-up plans for all COVID-19 positive cases. This would help in early diagnoses and initiation of the treatment and could be lifesaving in severe cardiac pathologies. In addition to this, the evaluation of large groups of patients divided by the categories vaccinated/nonvaccinated against COVID-19 would help to determine whether vaccine per se can offer protection in the occurrence of these types of complications.

## Figures and Tables

**Table 1:** Overview of the physiological parameters of the three patients at the first presentation.

		Case 1	Case 2	Case 3
BP (mmHg)	left arm	160/85	150/70	135/85
	right arm	130/85	145/75	135/80
HR (bpm)		110, irregular	62, regular	95, irregular
SpO_2_ (%)		98	95	98
Weight (Kg)		94	91	81
Height (cm)		176	181	177
AC (cm)		109	105	103
BMI (Kg/m^2^)		30.34	27.78	25.85
Smoking		no	yes, 11 PY	no

BP—blood pressure, HR—heart rate, SpO_2_—peripheral oxygen saturation pulse oximetry evaluated, AC—abdominal circumference, BMI—body mass index, PY—pack–year smoking cigarettes.

**Table 2:** Transthoracic echocardiographic findings of the three patients made in sinus rhythm.

	Case 1	Case 2	Case 3
IVS (cm)—PLAX	1.93	1.27	1.2
PLVW (cm)—PLAX	1.38	1.16	1.05
RV diameter (cm)—PLAX	4.1	3.7	3.59
LVTDV (ml/m^2^bs)—A4C	114.5	100.7	90.8
LVTSV (ml/m^2^bs)—A4C	63.8	54.07	50.9
LVEF (%)—A4C	43	46	44
RVTDV (ml/m^2^bs)—A4C	33.3	50.5	22.2
RVTSV (ml/m^2^bs)—A4C	15	20.6	13.5
LATSV (ml/m^2^bs)—A4C (*)	38.2	44.5	37.1
RATDS (ml/m^2^bs)—A4C (*)	22.7	50	10.4

IVS—interventricular septum, PLAX—parasternal long-axis view, PRV—right ventricle, LVW—posterior left ventricular wall, LVTDV—left ventricle tele-diastolic volume, bs—body surface, A4C—apical four-chamber view, LVTSV—left ventricle tele-systolic volume, LVEF—left ventricle ejection fraction, RVTDV—right ventricle tele-diastolic volume, RVTSV—right ventricle tele-systolic volume, (*)—regarding ventricular systole.

## Data Availability

Data will be provided upon written request.
